# YTHDF3 Mediates the Occurrence and Development of Breast Cancer by Regulating Glycolysis Through the mTOR–HIF1α–LHDA Axis

**DOI:** 10.1111/jcmm.71105

**Published:** 2026-04-26

**Authors:** ZiQian Liu, TengFei Jiang, Peng Li, Ke Dong, XiMei Wang, ChenChen Geng, XiaoDong Zhang, Dan Zuo, GuangHui Zhao

**Affiliations:** ^1^ Department of Medical Experimental Center, Qilu Hospital (Qingdao), Shandong University Qingdao China; ^2^ Department of General Surgery, Qilu Hospital (Qingdao), Shandong University Qingdao China; ^3^ Department of General Ultrasound, Qilu Hospital (Qingdao), Shandong University Qingdao China; ^4^ Medical Journal Editorial Department, Qingdao Hospital, University of Health and Rehabilitation Sciences (Qingdao Municipal Hospital), Qingdao Shandong China; ^5^ Peking University People's Hospital, Qingdao; Women and Children's Hospital Qingdao University Qingdao China

**Keywords:** breast cancer, glycolysis, mTOR–HIF1α–LDHA axis, YTHDF3

## Abstract

Breast cancer, a malignant tumour that frightens women, ruthlessly claims the lives of tens of thousands of women around the world every year. However, the mechanism by which YTHDF3 regulates the occurrence and development of breast cancer is still imperfect. In this study, we used bioinformatics to analyse the expression of YTHDF3 in breast cancer and verified it in clinical specimens. In addition, YTHDF3 knockdown breast cancer cell lines were used to determine the biological role of YTHDF3 in breast cancer through functional experiments such as CCK‐8 assay, transwell assay, clonal proliferation and cell cycle assay. Lactate analysis, seahorse assay, RT‐qPCR and Western blot were used to explore the downstream mechanism of YTHDF3, and rescue experiments were performed with small molecule activators to repeatedly confirm the downstream targets. Finally, YTHDF3 knockout breast cancer cell lines were used to establish cell line–derived xenograft (CDX) mouse models to further confirm the biological function of YTHDF3 in breast cancer and the possible regulatory mechanism. Our results showed that YTHDF3 was highly expressed in breast cancer cells and clinical tissues, and YTHDF3 enhanced the proliferation and migration ability of breast cancer. Mechanistically, YTHDF3 enhances the expression of HIF1α and LDHA and glycolysis by inducing the phosphorylation of mTOR, and finally promotes the occurrence and development of breast cancer. In addition, YTHDF3 can be used as a helpful biomarker in various cancers, including breast cancer. We aimed to elucidate the role of YTHDF3 in breast cancer development and provide evidence for improving the diagnosis and treatment of breast cancer. In this study, we elucidated that YTHDF3 regulates the glycolysis level, proliferation and migration of breast cancer through the mTOR–HIF1α–LDHA axis. Interference of the YTHDF3 expression is a potential target for breast cancer treatment, which provides strong evidence for improving diagnosis and treatment methods.

## 1 Introduction

The Annual Report to the Nation on the Status of Cancer is an update of rates for new cases and deaths as well as trends for the most common cancers in the United States (https://seer.cancer.gov/statfacts. gov/statfacts). According to statistics, there are estimated to be 297,790 new breast cancer cases in 2023, accounting for 15.2% of all new cancer cases, and 43,170 estimated new deaths in 2023, accounting for 7.1% of all cancer deaths. The 5‐year (2013–2019) relative survival rate of breast cancer is 90.8% [1, 2]. In most studies, breast cancer is divided into different molecular subtypes according to the different gene expression profiles, namely receptor‐positive: Luminal A, Luminal B, normal‐like and human epidermal growth factor receptor 2 (HER‐2) positive; Receptor‐negative: triple negative breast cancer (TNBC) or basal [3, 4, 5].

With the gradual deepening of breast cancer research, scientists have focused on the epigenetic level. Recent epigenetic studies have identified more than 170 different chemical modifications in RNA, which extensively regulate the behaviour and biological functions of RNA at the post‐transcriptional level [6]. N6‐methyladenosine (m6A) methylation is the most abundant post‐transcriptional modification on mRNA and plays a vital role in physiological and pathological processes [7]. There are three types of regulators of m6A modification: Writer: methyl transferases (MTC), which are responsible for catalysis, such as METTL3 [8], METTL14 [9] and WTAP [10]; Eraser: demethylases that remove methylation, such as FTO [11] and ALKBH5 [12]; Reader: directly recognizes and binds to the m6A site, so that m6A modified RNA can play a specific role, mainly including YTHDF1 [13], YTHDF2 [14] and YTHDF3 [15].

YTHDF3, a member of the YTHDF protein family, is responsible for mRNA translation and degradation [15, 16, 17]. In a recent study, Xiao et al. found that YTHDF3 promotes autophagy by recognizing the m6A modification site around the stop codon of FOXO3 mRNA. YTHDF3 also recruits eIF3a and eIF4B to promote FOXO3 translation and subsequently initiates autophagy [18]. Lin et al. found that YTHDF3 could enhance the stability of ZEB1 mRNA in TNBC in an M6A‐dependent manner, thereby positively regulating the migration, invasion and epithelial–mesenchymal transition (EMT) of TNBC cells [19]. LncRNA GAS5 in CRC directly binds to YAP, promoting its phosphorylation and ubiquitin‐mediated degradation, thereby weakening YAP‐mediated YTHDF3 transcription. YTHDF3 reversibly and selectively binds to m6A‐methylated GAS5, triggering its decay and forming a negative feedback loop [20]. Chang et al. showed that YTHDF3 overexpression was associated with breast cancer brain metastasis. Mechanistically, YTHDF3 enhanced the transcript expression of m6A, thereby promoting cancer cells‐brain microenvironment interaction and brain metastasis [21]. Although scientists have never stopped studying YTHDF3, most focus on its enhancing or weakening the expression of m6A transcript, and its role as a YTH domain‐containing protein is unknown [22].

The mechanistic target of rapamycin (mTOR) is a highly conserved serine/threonine kinase involved in cell metabolism, protein synthesis and cell death [23]. mTOR interacts with several proteins to form two distinct complexes: mTOR complex (mTORC)1 and mTORC2 [24]. mTOR can accept the regulation of upstream signals and switch to an activated state to perform functions such as stimulating protein synthesis, inhibiting catabolism and regulating cell death [25, 26, 27]. In cooperation with raptor, rictor, LST8 and mSin1, key components in mTORC1 or mTORC2, mTOR catalyses the phosphorylation of multiple targets such as ribosomal protein S6 kinase β‐1 (S6K1), eukaryotic translation initiation factor 4E binding protein 1 (4E‐BP1), Akt, protein kinase C (PKC) and type‐I insulin‐like growth factor receptor (IGF‐IR), thereby regulating protein synthesis, nutrients metabolism, growth factor signalling, cell growth and migration [28, 29]. Activation of the mTOR pathway promotes tumour growth and metastasis [30, 31, 32].

Cancer cells perceive hypoxia when they consume too much oxygen due to their hypermetabolism, so they make metabolic adaptations to compensate for the lack of oxygen supply [33, 34]. One major intracellular adaptation to severe hypoxia is the shift from oxidative phosphorylation to glycolysis [35, 36]. Hypoxia inducible factor 1 alpha (HIF1α) is a crucial transcription factor in cancer progression and target therapy in cancer [37]. Several studies have shown that the HIF1α expression increases in many cancers and plays an important role in cancer progression [38, 39, 40]. HIF1α induces the expression of many genes related to cancer cell growth and survival [41], angiogenesis [42], metastasis [43], cancer metabolism [44], cancer stem cell maintenance [45] and resistance to various cancer treatment modalities [46, 47]. In the final step of glycolysis, lactate dehydrogenase‐A (LDHA) converts pyruvate to lactate [48]. LDHA is a gene upregulated by HIF1α during cancer [49, 50]. It has been confirmed that the PI3K/Akt‐HIF1α pathway is involved in the downregulation of LDHA [51]. In another study, METTL3 can increase the transcription of LDHA via stabilizing the mRNA of HIF1α [50]. Therefore, we have reason to believe that the mTOR–HIF1α–LHDA axis can regulate glycolysis, thereby affecting the occurrence and development of cancer. However, the relationship between this pathway and YTHDF3 is still unclear and deserves further study.

This study aimed to reveal that YTHDF3 is highly expressed in breast cancer and that high levels of YTHDF3 promote breast cancer proliferation and migration. Mechanistic studies show that YTHDF3 regulates glycolysis through the mTOR–HIF1α–LHDA axis, mediates the occurrence and development of breast cancer and affects its prognosis. It is hoped that this result can provide a theoretical basis for designing new breast cancer treatment strategies.

## 2 Materials and Methods

### 2.1 Patient Samples and Cell Lines

Breast cancer and para‐cancerous tissues of 12 patients were collected from the Department of Breast Surgery, Qilu Hospital of Shandong University (Qingdao) from June 2021 to December 2022. After careful examination of the tissues by three pathologists, the patients were diagnosed with breast cancer. Patients who received any treatment (including surgery, chemotherapy, immunotherapy) before the surgery were excluded from the study. All patients signed informed consent. The study was approved by the Ethics Committee of Qilu Hospital of Shandong University (Qingdao). The study was in accordance with the Declaration of Helsinki.

The cell lines used in the study included human normal breast cell line MCF‐10A, human breast cancer cell line MCF‐7, MDA‐MB‐231, SKBR3, ZR751 and MDA‐MB‐453. MCF‐7, MDA‐MB‐231, SKBR3, ZR751 and MDA‐MB‐453 were purchased from Procell (Hunan, China). Cells were cultured in DMEM medium (Procell, China) supplemented with 10% FBS (Procell, China) and antibiotics (penicillin 100 U/mL, streptomycin 100 mg/mL) (Procell, China). For MCF‐10A, special medium produced by Procell was used. Cells were cultured at 37°C with 5% CO_2_.

### 2.2 Chemicals

Puromycin at a concentration of 10 mg/mL was obtained from MedChemExpress (Shanghai, China). The activator of the mTOR pathway, MHY1485, was obtained from MedChemExpress (Shanghai, China) at a concentration of 10 mM.

### 2.3 Lentiviral Construction and Transfection

Lentiviruses carrying short hairpin RNA (shRNA) against YTHDF3 were constructed by Genechem Company (Shanghai, China). The RNAi sequence targeting human YTHDF3 was 5′‐gaAGTCTGTTGTGGACTATAA‐3′ and 5′‐atAACCAATTACGGCATATTC‐3′. The negative control sequence was 5′‐TTCTCCGAACGTGTCACGT‐3′. Viruses were transfected with HitransG P according to the manufacturer's instructions.

### 2.4 Real‐Time Quantitative PCR

Total RNAs were extracted from cells using TRIzol reagent (Thermo Fisher, Shanghai, China) and reverse‐transcribed into cDNA using HiScript III All‐in‐one RT SuperMix Perfect for qPCR (Vazyme, Nanjing, China). Then, qPCR was performed using ChamQ Universal SYBR qPCR Master Mix (Vazyme, Nanjing, China). The primers were synthesized by Sangon Biotech (Shanghai, China) and Tsingke Biotech (Beijing, China). Gene‐specific primer sequences are detailed in Table 1.

**TABLE 1:** Primer sequences.

Gene name	Forward (5′–3′)	Reverse (5′–3′)
GAPDH	GGAGTCCACTGGCGTCTTCA	GTCATGAGTCCTTCCACGATACC
YTHDF3	GCTCCTAAGGCCCATGTTCTATC	TGGATCTGACATTGGTGGATAGC
HK1	CACATGGAGTCCGAGGTTTATG	CGTGAATCCCACAGGTAACTTC
HK2	GAGTTTGACCTGGATGTGGTTGC	CCTCCATGTAGCAGGCATTGCT
PFKL	AAGAAGTAGGCTGGCACGACGT	GCGGATGTTCTCCACAATGGAC
PKM	ATGTCGAAGCCCCATAGTGAA	TGGGTGGTGAATCAATGTCCA
LDHA	ATGGCAACTCTAAAGGATCAGC	CCAACCCCAACAACTGTAATCT
LDHB	GGACAAGTTGGTATGGCGTGTG	AAGCTCCCATGCTGCAGATCCA
HIF1α	TATGAGCCAGAAGAACTTTTAGGC	CACCTCTTTTGGCAAGCATCCTG

### 2.5 Western Blotting

Cells were washed with cold phosphate‐buffered saline (PBS, Procell, China) twice before protein extraction. Total proteins were extracted from cells using RIPA buffer (Solarbio, #R0010) supplemented with the PMSF (Solarbio, P0100) according to the manufacturer's instructions. 20–30‐μg proteins were loaded onto the 8%–12% SDS‐PAGE gel (Vazyme, Nanjing, China). Electrophoresis was performed at 80 V for 30 min and then at 120 V for 1 h. Proteins were then transferred to the PVDF membranes at 400 mA for 1 h. After blocking with 5% non‐fat milk for 1 h, membranes were incubated with primary antibodies overnight at 4°C. After TBST washing three times, the membranes were incubated with secondary antibodies for 1 h at room temperature. Primary antibodies used in the present study were listed as follows: GAPDH (ab313650, Abcam), HIF1 alpha (ab1, Abcam), mTOR (BM4182, Boster), p‐mTOR (BM4840, Boster), RICTOR (A03195‐1, Boster), RAPTOR (BM4438, Boster), AKT (BM4390, Boster), p‐AKT (BM4744, Boster), S6K (BM4240, Boster), p‐S6K (BM4141, Boster), LDHA (A1146, ABclonal), YTHDF3 (A8395, ABclonal), cyclin D1 (A11022, ABclonal), CDK4 (A23522, ABclonal), vimentin (PAB48967, Bioswamp), MMP9 (FNab05247, FineTest), LDHB (A5131, ABclonal), PKM1/2 (A27199PM, ABclonal), PFKL (A23687, ABclonal), snail (13099–1‐AP, Proteintech), E‐cadherin (PB9561, Boster), N‐cadherin (A01577‐3, Boster). HRP‐goat anti‐mouse IgG (AS003, ABclonal) and HRP‐goat anti‐rabbit IgG (AS014, ABclonal) were used as the secondary antibodies.

### 2.6 Immunohistochemistry

The tissues were first fixed in 4% paraformaldehyde for 48 h and then dehydrated. Next, the tissues were embedded in paraffin, sliced into 4‐μm sections and then dried in a 60°C oven for 2 h. The sections were deparaffinized and hydrated with xylene and gradient alcohol, respectively. 3% hydrogen peroxide was used to eliminate endogenous peroxidase activity. The sections were further repaired with Tris‐EDTA (pH = 9.0) and then blocked by BSA. Subsequently, the sections were incubated with primary antibody (anti‐YTHDF3, ABclonal, A8395, 1:500; anti‐HIF1α, Proteintech, 20,960–1‐AP, 1:500; anti‐LDHA, Proteintech, 2E2G6, 1:200; anti‐Ki67, Bioswamp, PAB44130, 1:400) overnight at 4°C, incubated with goat anti‐rabbit IgG at 37°C for 30 min, and stained with DAB reagent. At last, the sections were re‐stained with haematoxylin, dehydrated with gradient alcohol, mounted and photographed.

### 2.7 Cell Proliferation Assays

Cell proliferation was determined by cell Counting Kit‐8 (CCK‐8) assay. Approximately 3 × 10^3^ MDA‐MB‐231 and MCF‐7 cells were added to each well and seeded in 96‐well plates. 10 μL CCK‐8 (HY‐K0301, MCE, China) reagent was added to each well at 0, 24, 48, 72 and 96 h, respectively. After incubation for 2 h in an incubator at 37°C, the absorbance at 450 nm was determined.

### 2.8 Cell Invasion Assay

MDA‐MB‐231 cells and MCF‐7 cells' invasive ability was measured by transwell insert chambers (Corning, NY, USA) pre‐coated with Matrigel (Corning Inc.). In brief, 1 × 10^4^ cells were seeded into the upper chamber containing 200‐μL serum‐free medium, and 500‐μL culture medium with 20% FBS was added into the bottom chamber. After 24 h, invading cells were fixed with 4% paraformaldehyde and stained with 0.1% crystal violet. Cells were counted using a microscope for five fields randomly at 100× magnification.

### 2.9 Colony Formation Assays

Approximately 1 × 10^3^ cells were seeded in six‐well plates. After 2–3 weeks of treatment with puromycin, the cells were washed three times with PBS, fixed with 4% paraformaldehyde and stained with 0.1% crystal violet.

### 2.10 Cell Cycle Assays

1 × 10^5^ cells were seeded in a 6‐well plate for 48 h, and then cell cycle assays were performed. The procedures are briefly described: cells were collected by centrifugation at 12000 rpm for 5 min, and 70% cold ethanol was added to the cells dropwise. Cells were then fixed at 4°C overnight. Next, wash the cells with 1 × PBS twice. After staining the cells with propidium iodide (PI) antibody for 30 min, the cell cycle was measured by flow cytometry.

### 2.11 Lactic Acid Assays

Lactic acid was detected by L‐lactic acid (LA) colorimetric test kit (E‐BC‐K318‐M, Elabscience, China) according to the reagent instructions. Before detection, enzyme working solutions and standards with different concentrations were prepared. A total of 10^6^ cells were added to PBS for homogenization. After homogenization, the samples were centrifuged at 10,000 × g for 10 min at 4°C, and the supernatant was removed for detection. Data for each well were normalized to protein concentration as determined using the BCA Protein Assay kit (E‐BC‐K318‐M, Elabscience, China).

### 2.12 Glycolysis Stress Test

Extracellular acidification rate (ECAR) was measured on a Seahorse XFe24 extracellular flux analyser (Agilent) following the manufacturer's instructions. In brief, 4 × 10^5^ cells per well were seeded in cell culture microplates (Sigma‐Aldrich). After reaching 80%–90% confluency, cells were equilibrated for 1 h in an XF assay medium supplemented with 2 mM glutamine in a non‐CO_2_ incubator. ECAR was monitored at baseline, and sequential injections of glucose (10 mM), oligomycin (1 μM) and 2‐deoxy‐glucose (2‐DG) (50 mM) were used. Data for each well were normalized to protein concentration as determined using the BCA Protein Assay kit (E‐BC‐K318‐M, Elabscience, China) after measurement on the XFe24 machine.

### 2.13 Cell Mitochondrion Stress Test

The Agilent Seahorse XFp Cell Mito Stress Test measures key parameters of mitochondrial function by directly measuring the oxygen consumption rate (OCR) of cells on the Seahorse XFp Extracellular Flux Analysers. It is a plate‐based live cell assay that enables monitoring OCR in real time. OCR was measured on a Seahorse XFe24 extracellular flux analyser (Agilent) following the manufacturer's instructions. In brief, 4 × 10^5^ cells per well were seeded in cell culture microplates (Sigma‐Aldrich). After reaching 80%–90% confluency, cells were equilibrated for 1 h in an XF assay medium supplemented with 1 mM sodium pyruvate, 2 mM glutamine and 10 mM glucose in a non‐CO_2_ incubator. OCR was monitored at baseline, and sequential injections of oligomycin (1.5 μM), FCCP (1 μM), Rotenone/antimycin A (Rot/AA) (0.5 μM) were used. Data for each well were normalized to protein concentration as determined using the BCA Protein Assay kit (E‐BC‐K318‐M, Elabscience, China) after measurement on the XFe24 machine.

### 2.14 Mitochondrial Respiratory Chain Complex I Activity Test

The enzymatic activity of complex I was detected using test kit (BC0515, Solarbio, China). Complex I is also known as NADH‐CoQ Reductase or NADH Dehydrogenase. It is widely present in the mitochondria of animals, plants, microorganisms and cultured cells, and is the largest protein complex in the inner mitochondrial membrane. This enzyme catalyses the transfer of a pair of electrons from NADH to CoQ, and can also reduce O_2_ to form O_2_−. It is the main site for the production of O_2_− in the respiratory electron transfer chain. Measuring the activity of this enzyme can not only reflect the state of the respiratory electron transfer chain (ETC) but also reflect the generation state of reactive oxygen species (ROS). Complex I can catalyse the dehydrogenation of NADH to form NAD+. The oxidation rate of NADH at 340 nm can be measured to calculate the size of the enzyme activity.

### 2.15 Nomogram and Survival Analysis

RNA‐seq data and clinical information on breast cancer were downloaded from UCSC Xena (https://xenabrowser.net/datapages/). Column Nomograms were constructed using YTHDF3 mRNA expression levels, age, T stage, N stage, M stage and tumour stage data of breast cancer patients through the RMS package in R language. Patients were classified into high and low YTHDF3 expression groups using the median YTHDF3 expression level as the threshold. The difference in survival prognosis between the two groups was then analysed using the survival package.

### 2.16 In Vivo

Female BALB/c nude mice (4–6 weeks of age, 18–20 g) were purchased from Beijing Vital River Laboratory Animal Technology Co. Ltd. (Beijing, China). All the animal experiments were conducted with the approval of the Institutional Animal Care and Use Committee of Shandong University Qilu Hospital. 5 × 10^6^ MDA‐MB‐231 cells transduced with shNC or shYTHDF3 were resuspended in 100 μL PBS and subcutaneously injected into the mice (5 animals/group). The length and width of tumours were measured with a vernier caliper every 7 days. Tumour volume was calculated by the formula: *V* = π/6 × length×width^2^. After 9 weeks, mice were sacrificed, and the weight of tumours was detected. Xenografts were collected for RT‐qPCR, immunohistochemistry staining, and western blot analysis.

### 2.17 Statistical Analysis

All experiments were repeated three or more times. All quantitative data are presented as the means ± SDs. We used a standard two‐tailed unpaired *t*‐test for the statistical analysis of the two groups. Two‐way ANOVA was used for statistical analysis of multiple groups of data. *p* < 0.05 was considered to indicate statistical significance. *p < 0.05, **p < 0.01, ***p < 0.001, ****p < 0.0001, ns represents no significance.

## 3 Results

### 3.1 YTHDF3 Is Highly Expressed in Breast Cancer Tissues and Cells

**FIGURE 1:**
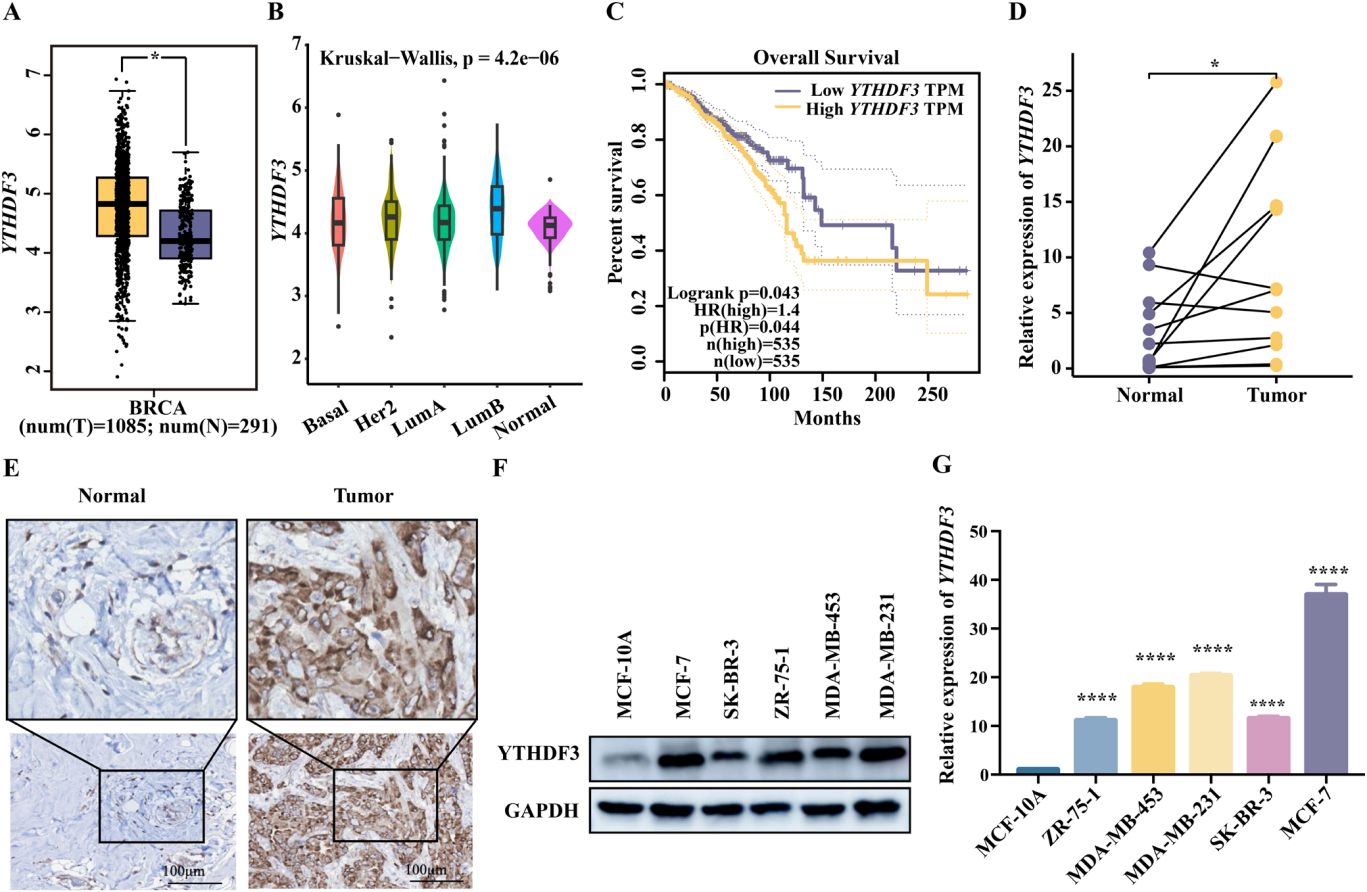
YTHDF3 is overexpressed in breast cancer tissues and cells. (A) YTHDF3 expression in breast cancer tissues and adjacent normal tissues in TCGA database; (B) YTHDF3 expression in different types of breast cancer in TCGA database; (C) Kaplan–Meier curves of OS in the TCGA dataset of all breast cancer patients with high and low YTHDF3 expression; (D) YTHDF3 expression in paired breast cancer tissues and adjacent normal tissues; (E) YTHDF3 expression of breast cancer and para‐cancerous tissues was evaluated by immunohistochemistry, ×100; (F) YTHDF3 protein expression in normal human breast cell line MCF‐10A and breast cancer cell lines MCF‐7, MDA‐MB‐231, SK‐BR‐3, ZR‐75‐1 and MDA‐MB‐453; (G) Relative YTHDF3 mRNA expression in normal human breast cell line MCF‐10A and breast cancer cell lines MCF‐7, MDA‐MB‐231, SK‐BR‐3, ZR‐75‐1 and MDA‐MB‐453.

To confirm the proteins among m6A ‘reader’ proteins that may drive breast carcinogenesis, we first explored the expression of m6A ‘reader’ proteins in the TCGA‐BRCA database (Figure 1A and Figure S1). The results showed that the expression of YTHDF3 in breast cancer tissues was significantly higher than that in normal controls (Figure 1A). Moreover, the expression of YTHDF3 in breast cancer tissues of different types of breast cancer patients was significantly higher than that in the normal control group (Figure 1B). We also examined the effect of YTHDF3 on the prognosis of breast cancer patients. Kaplan–Meier analysis showed that elevated YTHDF3 was associated with shortened overall survival (OS), a marker of poor prognosis in breast cancer, indicating that patients with high YTHDF3 expression had a poor prognosis (Figure 1C). Similar results were obtained by RT‐qPCR and immunohistochemical staining in paired patient breast cancer tissues and adjacent normal tissues (Figure 1D,E). Subsequently, we further investigated the expression of YTHDF3 in MCF‐10A, MCF‐7, SKBR3, ZR751, MDA‐MB‐453 and MDA‐MB‐231 cell lines. Western blot and RT‐qPCR results showed that YTHDF3 expression was higher in each breast cancer cell line than in MCF‐10A (Figure 1F,G). Taken together, these results demonstrate that YTHDF3 was dysregulated in breast cancer and that high expression of YTHDF3 was associated with poor outcomes in patients with breast cancer.

### 3.2 YTHDF3 Knockdown Inhibits Proliferation and Migration of Breast Cancer Cells

**FIGURE 2:**
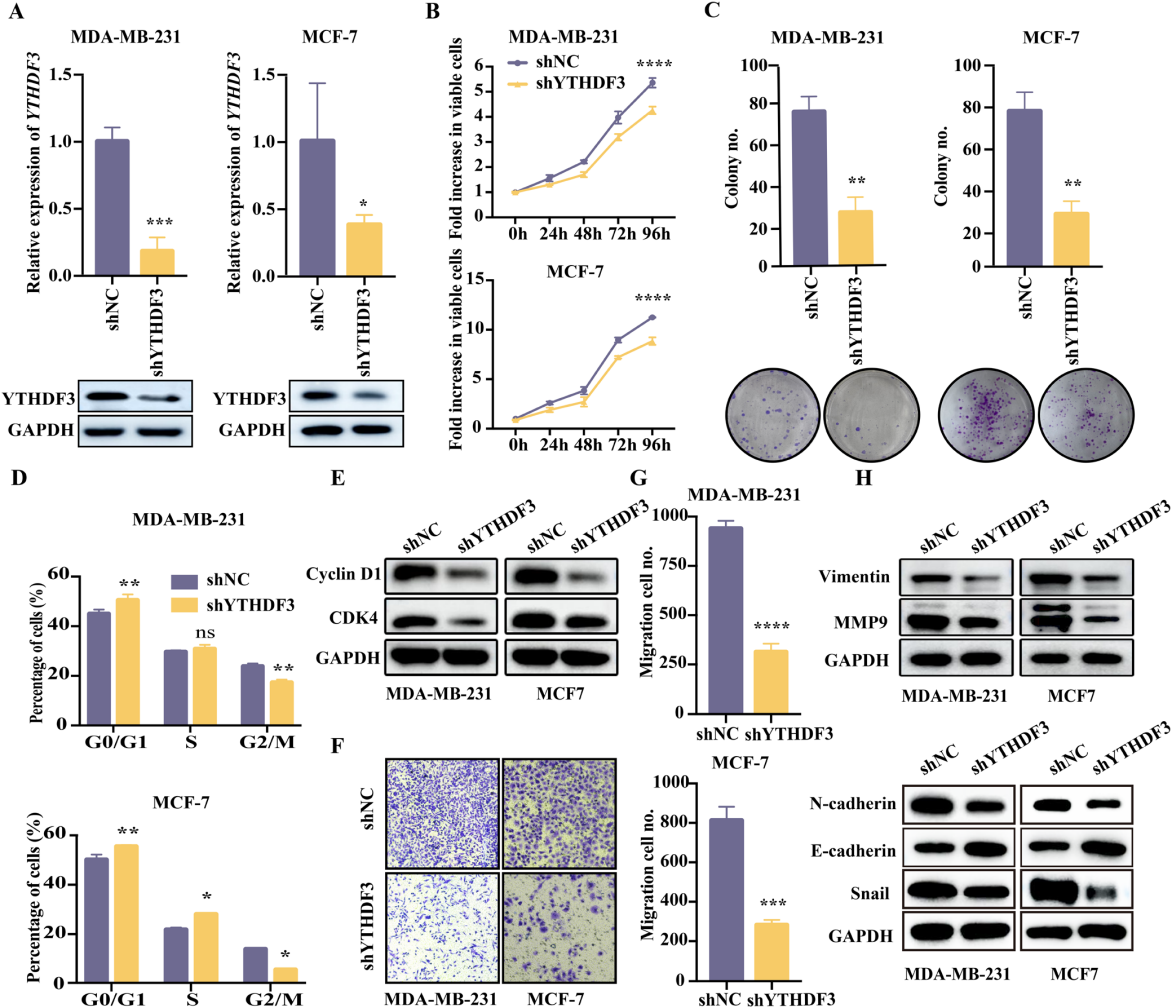
Silence of YTHDF3 restrain cell proliferation and migration of breast cancer cells and arrested the cell cycle. (A) Relative expression of YTHDF3 mRNA and protein after transfection of shNC and shYTHDF3 in MDA‐MB‐231 and MCF‐7 cells; (B) the growth ability of MDA‐MB‐231 and MCF‐7 cells transfected with shNC and shYTHDF3 was detected by CCK‐8 assay; (C) colony formation assay was used to detect the proliferation ability of MDA‐MB‐231 and MCF‐7 cells in shNC and shYTHDF3 groups; (D) the cell cycle distribution of MDA‐MB‐231 and MCF‐7 cells in shNC and shYTHDF3 groups was detected by flow cytometry; (E) expression of cyclin D1 and CDK4 in shNC and shYTHDF3 in MDA‐MB‐231 and MCF‐7 cells; (F, G) transwell invasion assay was used to detect the migration ability of breast cancer cells in the shNC group and the shYTHDF3 group in MCF‐7 and MDA‐MB‐231 cells; (H) the expression of vimentin, MMP9, E‐cadherin, N‐cadherin and snail in shNC and shYTHDF3 in MDA‐MB‐231 and MCF‐7 cells.

YTHDF3 shRNA was transfected into breast cancer cell lines MDA‐MB‐231 and MCF‐7 by lentivirus to establish stable YTHDF3 low‐expression cell lines. After 48 h of virus transfection, the successfully transfected cells were selected with 2 μg/mL of puromycin. YTHDF3 mRNA and protein expression were detected to verify the knockdown efficiency, and the results showed that the expression of YTHDF3 was significantly inhibited by shRNA in MDA‐MB‐231 and MCF‐7 cells (Figure 2A and Figure S2A). We then performed CCK‐8 cell proliferation, clone formation, cell cycle and transwell assays using scramble shRNA‐transfected MDA‐MB‐231 and MCF‐7 cells (shNC) and shYTHDF3‐transfected cancer cells. CCK‐8 results showed that compared with the shNC group, the proliferation of the shYTHDF3 group was significantly inhibited after 48 h of incubation (Figure 2B and Figure S2B). The colony formation assay showed that the number of clones formed by shYTHDF3 cells was significantly reduced compared with the shNC group (Figure 2C and Figure S2C,D). In addition, the effect of YTHDF3 on cell cycle was investigated, and the results showed that G0/G1 cell cycle arrest occurred in the shYTHDF3 group compared with the shNC group (Figure 2D). Next, we studied the expression of cyclin D1 and cyclin dependent kinase 4 (CDK4), which are related to the cell cycle. The results showed that compared with the shNC group, the expression of cyclin D1 and CDK4 in the shYTHDF3 group was significantly reduced (Figure 2E). We investigated the role of YTHDF3 in breast cancer cell invasion. Transwell assay showed that knockdown of YTHDF3 effectively inhibited the migration of MDA‐MB‐231 and MCF‐7 cells (Figure 2F,G and Figure S2E,F). Subsequently, we measured the levels of key proteins associated with tumour metastasis and EMT, such as vimentin, matrix metalloproteinase 9 (MMP9), E‐cadherin, N‐cadherin and snail. Western blotting showed that compared with the shNC group, the levels of vimentin, MMP9, N‐cadherin and snail proteins were decreased in the shYTHDF3 group, while the level of E‐cadherin was increased (Figure 2H). Therefore, inhibition of YTHDF3 expression in MCF‐7 and MDA‐MB‐231 cells significantly reduced the proliferation, cloning and migration ability of breast cancer cells.

### 3.3 Silencing YTHDF3 Caused a Decrease in Lactate Levels and Affected Glycolysis

**FIGURE 3:**
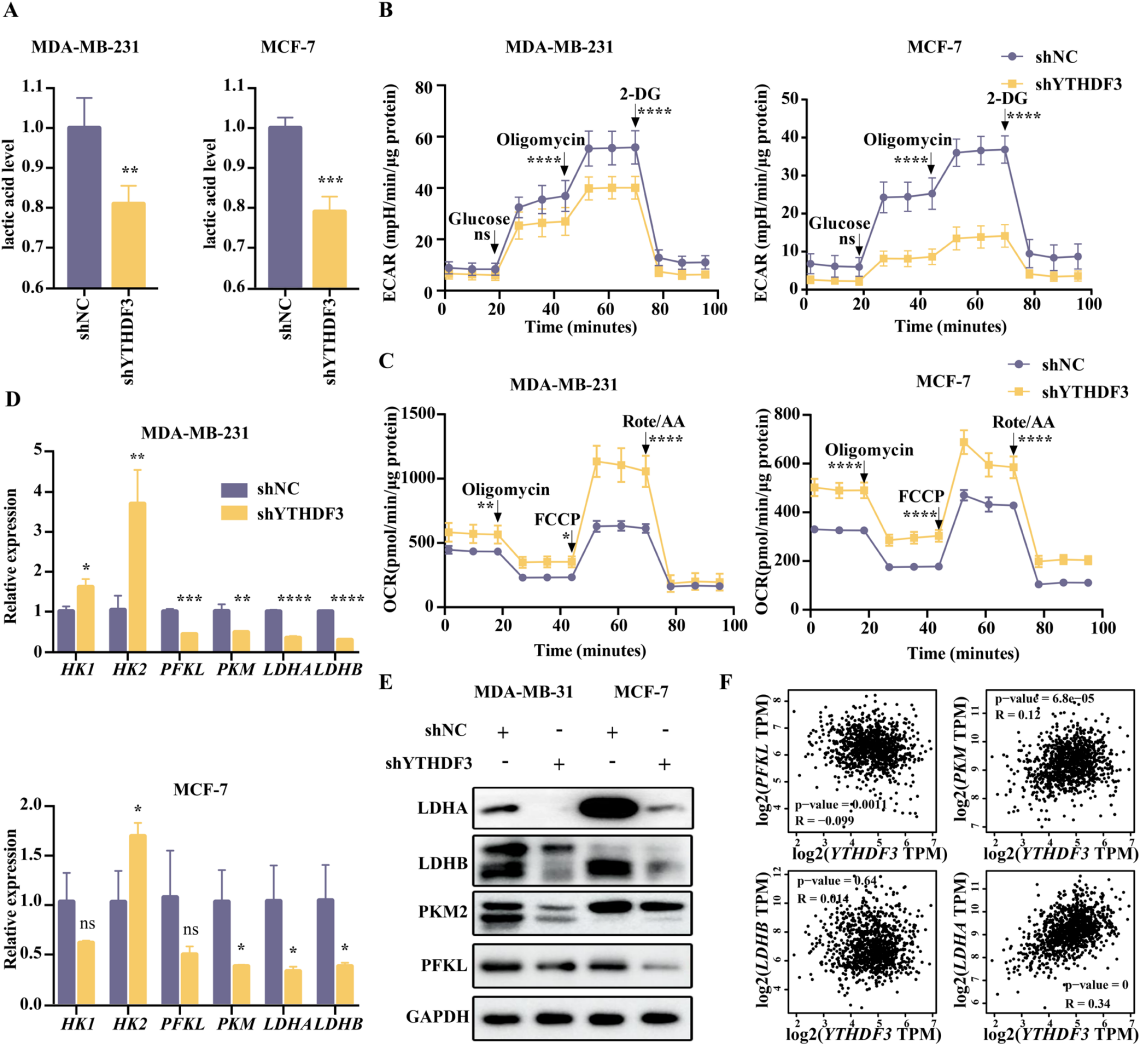
Silencing YTHDF3 caused a decrease in lactate levels and affected glycolysis. (A) Lactate levels in shNC and shYTHDF3 groups in MDA‐MB‐231 and MCF‐7 cells; (B) extracellular acidification rate (ECAR) in shNC and shYTHDF3 groups in MDA‐MB‐231 and MCF‐7 cells measured by a seahorse instrument; (C) oxygen consumption rate (OCR) in shNC and shYTHDF3 groups in MDA‐MB‐231 and MCF‐7 cells measured by a seahorse instrument; (D) relative expression levels of hexokinase‐1 (HK1), hexokinase‐2 (HK2), PFKL, PKM, LDHA and LDHB in shNC and shYTHDF3 groups in MDA‐MB‐231 and MCF‐7 cells; (E) protein expression levels of PFKL, PKM, LDHA and LDHB in shNC and shYTHDF3 groups in MDA‐MB‐231 and MCF‐7 cells; and (F) the correlation between YTHDF3 and PFKL, PKM, LDHA and LDHB was predicted using the GEPIA website.

In Figure 2, we further demonstrate that YTHDF3 levels can influence cell growth, and that cancer cell growth depends on glycolysis [52]. The first thing we looked at was lactic acid. We examined lactate levels in shNC and shYTHDF3 groups and found that intracellular lactate levels were significantly decreased after YTHDF3 knockdown (Figure 3A). This led us to wonder if YTHDF3 also affects cellular metabolism, so we shifted our focus to the metabolic effects of YTHDF3. To assess the functional role of YTHDF3 in glycolysis, the extracellular acidification rate (ECAR) and OCR were measured using a seahorse instrument. Under YTHDF3 knockdown conditions, ECAR was significantly reduced in MDA‐MB‐231 and MCF‐7 cells, which were mainly determined by the production of lactate from glycolysis (Figure 3B). However, OCR outside MDA‐MB‐231 and MCF‐7 cells was significantly increased (Figure 3C and Figure S3A,B). In other words, YTHDF3 promotes glycolytic metabolism in breast cancer cells. Up to this point, we were able to determine that downregulation of YTHDF3 levels affected intracellular glycolysis. Therefore, several key enzymes in glycolysis were selected and their relative expression levels before and after YTHDF3 knockdown were detected by RT‐qPCR (Figure 3D). The results showed that the decrease of YTHDF3 level caused the decrease of phosphofructokinase‐liver type (PFKL), pyruvate kinase M (PKM), lactate dehydrogenase A (LDHA) and lactate dehydrogenase B (LDHB) expression. We then formally confirmed this phenomenon at the protein level (Figure 3E and Figure S3C). We predicted the correlation between YTHDF3 and PFKL, PKM, LDHA and LDHB through the gene expression profiling interactive analysis (GEPIA) website (Figure 3F). Combined with qPCR and western blot results of cell lines and correlation analysis, LDHA expression was positively correlated with YTHDF3 expression and the *p*‐value was significant.

### 3.4 YTHDF3 Regulates the Glycolysis Level of Breast Cancer Through the mTOR–HIF1α–LDHA Axis

**FIGURE 4:**
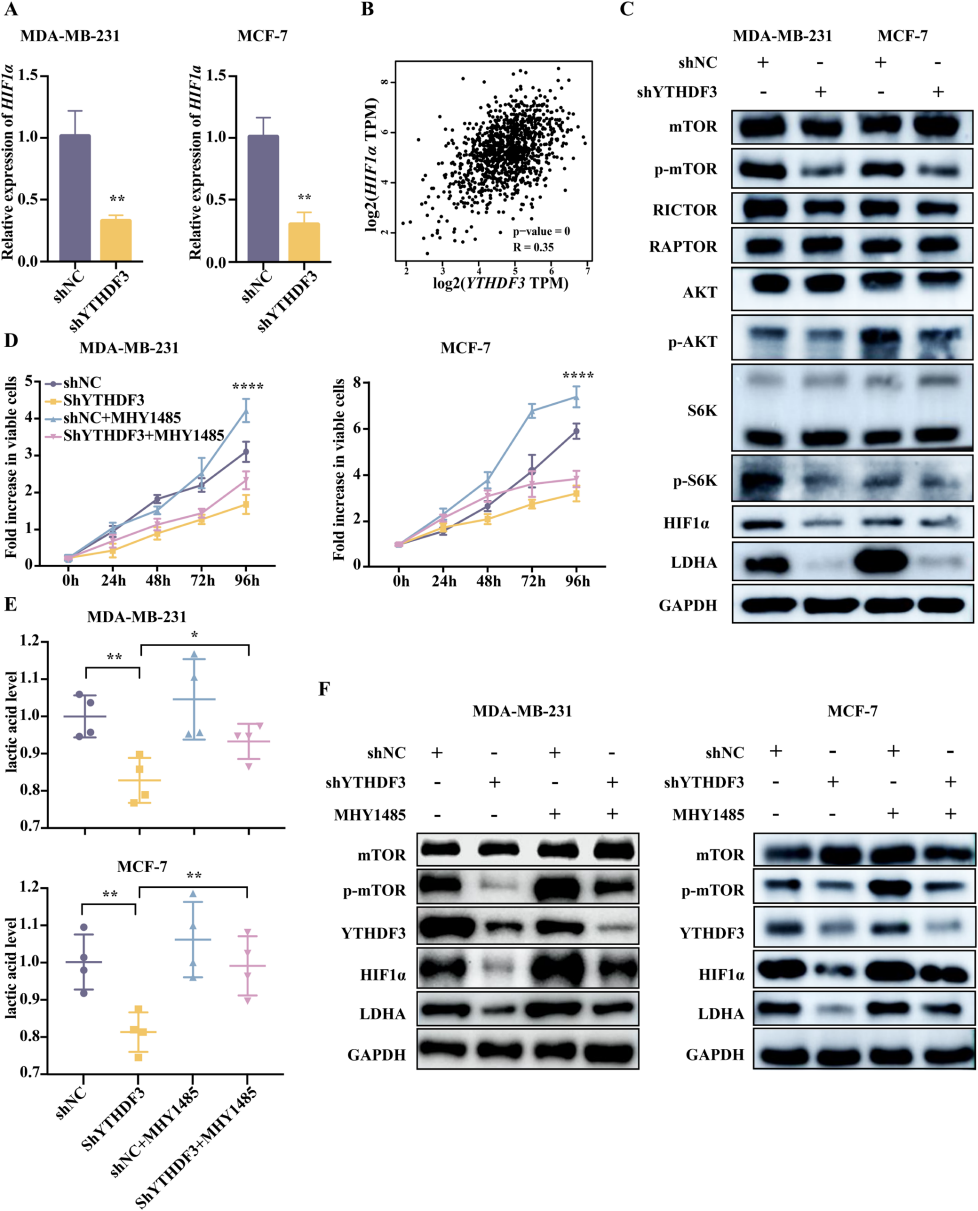
YTHDF3 regulates the glycolysis level of breast cancer through the mTOR–HIF1α–LDHA axis. (A) Relative mRNA expression of HIF1α in shNC and shYTHDF3 groups in MDA‐MB‐231 and MCF‐7 cells; (B) the correlation between YTHDF3 and HIF1α was predicted using the GEPIA website; (C) expression of key proteins in the mTOR–HIF1α–LDHA axis in shNC and shYTHDF3 groups in MDA‐MB‐231 and MCF‐7 cells; (D) after adding activator MHY1445, the growth ability of cancer cells in shNC and shYTHDF3 groups was detected by CCK‐8 assay; (E) the lactate level of cancer cells in shNC and shYTHDF3 groups treated with activator MHY1485; and (F) expression of proteins of cancer cells in shNC and shYTHDF3 groups treated with activator MHY1485.

It is well known that many cancer cells take up glucose in large quantities and produce lactate through LDHA due to tumour hypoxia [53]. Previous studies have shown that mTOR–HIF1α plays a critical role in cancer development and HIF1α directly regulates LDHA expression as a transcription factor [54]. Therefore, it is reasonable to further explore the regulation of YTHDF3 on glycolytic levels in breast cancer through the mTOR–HIF1α–LDHA axis. The results of RT‐PCR and bioinformatics analysis showed that the expression of HIF1α was significantly decreased in breast cancer cells with YTHDF3 knockdown compared with the control group (Figure 4A,B). Then, we selected key proteins on the mTOR–HIF1α–LDHA axis for Western blot analysis, and the results showed that YTHDF3 expression changes the phosphorylation level of mTOR and the expression levels of HIF1α and LDHA (Figure 4C and Figure S3C). To further clarify the role of mTOR phosphorylation in breast cancer proliferation and its regulatory effect on HIF1α‐LHDA‐lactate, we added MHY1485, an activator of mTOR phosphorylation, to YTHDF3 knockdown. The results showed that MHY1485 treatment rescued the proliferation inhibition caused by YTHDF3 interference alone (Figure 4D) and increased its lactate level, indicating the rescue of glycolysis level (Figure 4E). Mechanologically, MHY1485 treatment also increased the expression levels of HIF1α and LDHA (Figure 4F). Taken together, these results provide bidirectional evidence that YTHDF3 regulates the glycolysis level and promotes breast cancer proliferation through the mTOR–HIF1α–LDHA axis.

### 3.5 YTHDF3 Regulates Breast Cancer Development Through mTOR–HIF1α–LDHA Axis In Vivo

**FIGURE 5:**
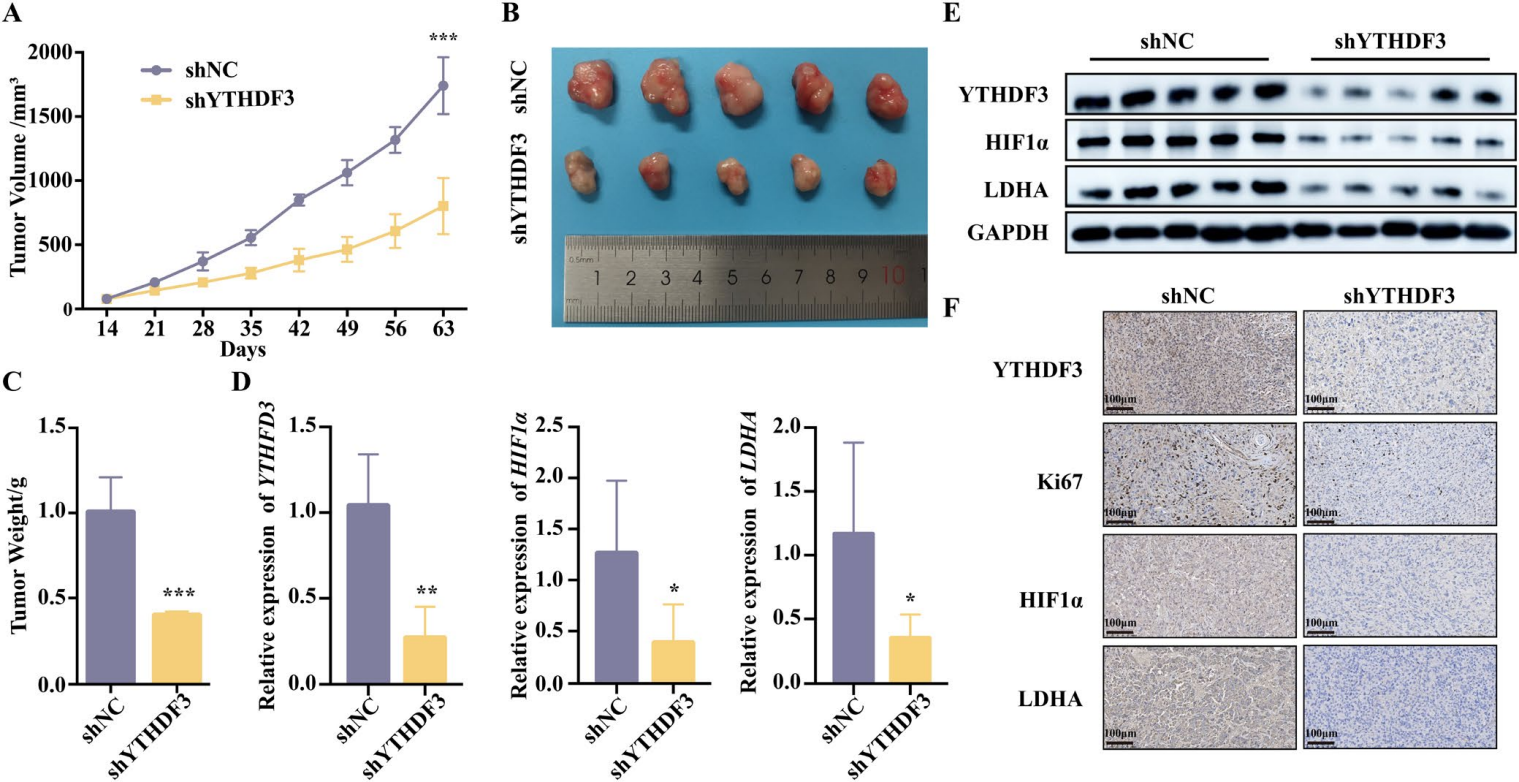
YTHDF3 regulates breast cancer development through the mTOR–HIF1α–LDHA axis in vivo. (A) Tumour volumes in shNC and shYTHDF3 groups on the indicated days; (B) representative tumour images and (C) tumour weights in shNC and shYTHDF3 groups on day 63; (D) mRNA and (E) protein expression levels of YTHDF3, HIF1α and LDHA in the tumours in shNC and shYTHDF3 groups; (F) Immunohistochemical images of YTHDF3, Ki67, HIF1α and LDHA in tumours in the shNC and shYTHDF3 groups, ×100.

To further investigate whether YTHDF3 affects cancer cell growth in vivo through the mTOR–HIF1α–LDHA axis, breast cancer cell line MDA‐MB‐231 with stable knockdown of YTHDF3 was selected and inoculated into immunodeficient mice. After 9 weeks, the mice were sacrificed, the tumour size was observed, and the tumour volume and weight were measured (Figure 5A–C). Consistent with our conclusion, the knockdown of YTHDF3 significantly inhibited tumour growth in vivo. Then, the removed mouse tumours were divided into triplicate. Tumour mRNA and protein were extracted to verify the expression of YTHDF3, HIF1α and LDHA. The results showed that the mRNA and protein expression levels of YTHDF3, HIF1α and LDHA in the tumours of the shYTHDF3 group were significantly decreased (Figure 5D,E). The remaining tumour tissues were taken for immunohistochemical staining. The results showed that compared with the control group, the shYTHDF3 group had fewer Ki67 (proliferation marker) positive cells in tumours, and also fewer YTHDF3, HIF1α and LDHA positive cells (Figure 5F). Our in vivo study further confirmed that YTHDF3 regulated glycolytic level through mTOR–HIF1α–LDHA axis to promote breast cancer development.

### 3.6 YTHDF3 May Be a Potent Marker of Prognosis in Multiple Cancers

**FIGURE 6:**
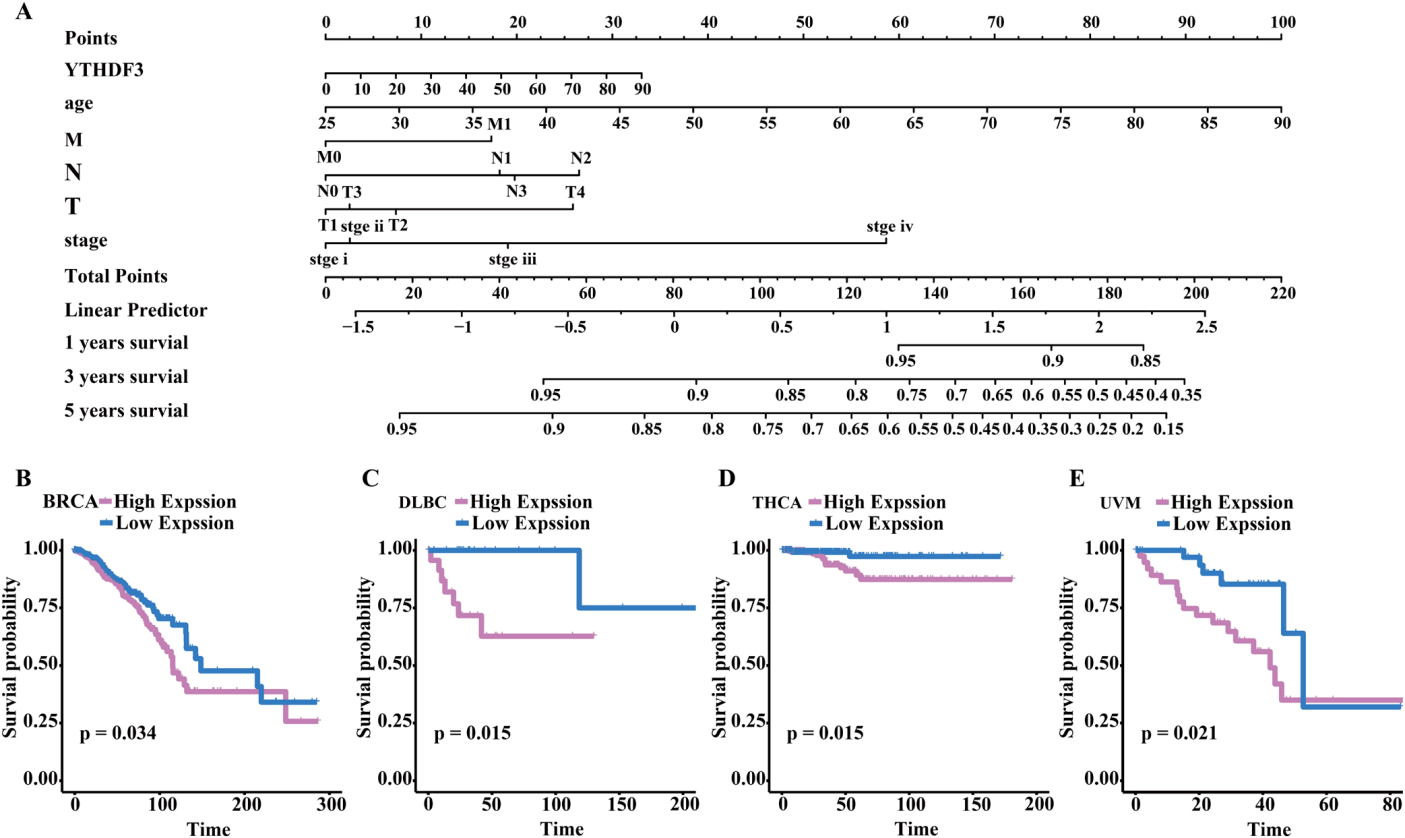
YTHDF3 is associated with prognosis in several cancers. (A) Column nomogram was constructed using YTHDF3 expression levels, age, T stage, N stage, M stage and tumour stage data of breast cancer patients; (B–E) survival analysis software package was used to analyse the survival prognosis difference between patients with high and low YTHDF3 expression in BRCA, DLBC, THCA and UVM.

Both in vitro and in vivo experiments demonstrated that YTHDF3 promotes breast cancer progression through the mTOR–HIF1α–LDHA axis, and we next hoped to suggest that YTHDF3 could be used as a marker for predicting the prognosis of breast cancer through clinical data. First, we constructed a Lenorm model that can guide the treatment of breast cancer patients by using the data of YTHDF3 mRNA expression level, age, T stage, N stage, M stage and tumour stage. We found that high YTHDF3 expression, increasing age, and higher scores in patients with advanced stages predicted poorer survival in breast cancer patients (Figure 6A). In addition, we analysed the correlation between YTHDF3 mRNA expression levels and survival rates in various cancer types. We found that high YTHDF3 expression may be a risk factor or protective factor. For example, in breast cancer (BRCA), diffuse large B‐cell lymphoma (DLBC), thyroid cancer (THCA) and uveal tract melanoma (UVM), high expression of YTHDF predicted poor survival (Figure 6B–E), while in colonic adenocarcinoma (COAD), acute myeloid leukaemia‐like (LAML), kidney renal clear cell carcinoma (KIRC) and mesothelioma (MESO), high expression of YTHDF3 predicted good survival (Figure S4A–D). In conclusion, YTHDF3 can be used as an effective marker to predict the survival of cancer patients in a wide range of cancer types.

## 4 Discussion

The Warburg effect of tumour cells is a complex process regulated by various molecular networks. The balance between oxidative phosphorylation and glycolysis is the key to controlling the Warburg effect [55]. YTHDF3 is a member of the YTHDF protein family and plays multiple roles in physiological and pathological settings as a ‘reader’ of m6A methylation [56]. In this study, we found that YTHDF3 was highly expressed in cancer tissues and cancer cell lines, and specific knockdown of YTHDF3 delayed tumour cell proliferation and reduced tumour cell migration ability. To further explore the mechanism of this phenomenon, YTHDF3 deficiency reduced the phosphorylation level of mTOR and the expression levels of HIF1α and LDHA, resulting in the decreased glycolytic ability of breast cancer cells, thereby impairing the proliferation and migration of tumour cells. The current study has further enriched the molecular regulatory network of the occurrence and development of breast cancer, and provided new clues and effective markers for the diagnosis and treatment of breast cancer.

Previous studies have focused on YTHDF3 as an m6A methylation ‘reader’ to bind and recognize the methylation site so that m6A‐modified RNA can exert its specific biological functions. For example, YTHDF3 promotes HCC metastasis in digestive system cancers by maintaining ZEB1 mRNA stability through an m6A‐dependent mechanism [57]. In addition, Ni et al. revealed that the long noncoding RNA GAS5‐YAP‐YTHDF3 axis forms a feedback loop in CRC [20]. Similarly, in ocular melanoma, YTHDF3 also contributed to its progression by promoting CTNNB1 mRNA translation in an m6A‐dependent manner [58]. Here, we found that the expression of YTHDF3 could directly affect the phosphorylation of mTOR to promote the glycolysis level of breast cancer cells and ultimately promote the proliferation and migration of breast cancer cells. In addition, in the animal model, it was also well verified that the occurrence and development of breast cancer cells were significantly inhibited after knocking down YTHDF3. Therefore, our findings reveal the role and mechanism of YTHDF3 in breast cancer development.

In the 1920s, Otto Warburg and colleagues observed that cancer cells preferred glycolysis to meet the biosynthetic and bioenergetic demands of uncontrolled growth compared with surrounding tissues and to convert glucose to lactate at high rates for tumour progression [59]. We found that the expression of YTHDF3 could affect the proliferation and migration of breast cancer cells, and further confirmed that YTHDF3 could directly affect the production of lactate and the level of glycolysis. In driving the glycolysis process, several key rate‐limiting enzymes (HK1, HK2, PFKL, PKM, LDHA, LDHB, etc.) play important roles, and cancer cells tend to metabolize more pyruvate to lactate instead of the tricarboxylic acid cycle. In turn, cancer cells transport lactate out of the cells through the mono‐carboxylic acid transporter (MCT), and the transported lactate will change the pH value of the tumour microenvironment (TME) and form an immunosuppressive microenvironment [60]. In the isoenzyme of LHD, cancer cells mainly express LHDA [61]. Therefore, we verified by altering the expression of YTHDF3, and found that YTHDF3 can up‐regulate the expression of LDHA. As an important signal transduction intermediate, mTOR participates in various intracellular signalling pathways, including the regulation of HIF1α. HIF1 is activated by mTOR in cancer cells and mediates metabolic alterations that drive cancer progression and resistance to therapy [54]. As our predictive gene, we further explored the role of mTOR–HIF1α on LDHA‐mediated lactate metabolism. We found that knockdown of YTHDF3 significantly reduced the activation of mTOR, the expression of HIF1α and LDHA, and the level of lactate in the supernatant, which were rescued by MHY1485, an mTOR activator. These results support that YTHDF3 promotes glycolysis and contributes to breast cancer development through the mTOR–HIF1α–LHDA axis.

Our results suggest that YTHDF3 is involved in breast cancer cell proliferation and migration through the activation of mTOR, which up‐regulates HIF1α and LDHA. Although the role of YTHDF3 in regulating mTOR activation still deserves further investigation, the findings of this study still contribute to a better understanding of the regulatory mechanism of breast cancer development and provide a potential target for improving the diagnosis and treatment of breast cancer.

## Author Contributions

**ZiQian Liu:** conceptualization (equal), validation (equal), writing – original draft (equal). **TengFei Jiang:** data curation (equal), validation (equal). **Peng Li:** data curation (equal), resources (equal). **Ke Dong:** data curation (equal), investigation (equal). **XiMei Wang:** formal analysis (equal), project administration (equal). **ChenChen Geng:** resources (equal), software (equal). **XiaoDong Zhang:** writing – original draft (equal), writing – review and editing (equal). **Dan Zuo:** validation (equal), writing – review and editing (equal). **GuangHui Zhao:** funding acquisition (equal), writing – review and editing (equal).

## Funding

This work was supported by the Shandong University of Qilu Hospital (Qingdao) Internal Medicine Research Initiation Fund Youth Project (Grant QDKY2022QN01), and the Natural Science Foundation of Shandong Province (Grant ZR2022MH084).

## Conflicts of Interest

The authors declare no conflicts of interest.

## Supporting information


**Figure S1:** Expression of m6A ‘readers’ in cancer and adjacent tissues in the TCGA database. (A) YTHDF1 expression in breast cancer tissues and adjacent normal tissues in the TCGA database; (B) YTHDF2 expression in breast cancer tissues and adjacent normal tissues in the TCGA database; (C) YTHDC1 expression in breast cancer tissues and adjacent normal tissues in the TCGA database; (D) YTHDC2 expression in breast cancer tissues and adjacent normal tissues in the TCGA database.
**Figure S2:** Silence of YTHDF3 restrains cell proliferation and migration of breast cancer cells and arrested the cell cycle. (A) Relative expression of YTHDF3 mRNA and protein after transfection of shNC and shYTHDF3‐2# in MDA‐MB‐231 and MCF‐7 cells; (B) the growth ability of MDA‐MB‐231 and MCF‐7 cells transfected with shNC and shYTHDF3‐2# was detected by the CCK‐8 assay; (C, D) colony formation assay was used to detect the proliferation ability of MDA‐MB‐231 and MCF‐7 cells in shNC and shYTHDF3‐2# groups; (E, F) transwell invasion assay was used to detect the migration ability of breast cancer cells in the shNC group and the shYTHDF3‐2# group in MCF‐7 and MDA‐MB‐231 cells.
**Figure S3:** Supplementary experiments of YTHDF3. (A) The results of non‐mitochondrial, basal, maximal, proton leak, ATP production respiration and spare respiratory capacity OCR were normalized by protein amount (μg protein); (B) the mitochondrial complex I activity in the shNC group and the shYTHDF3 group; (C) the expression of YTHDF3 protein in shNC and shYTHDF3 groups of MDA‐MB‐231 and MCF‐7 cell lines; this is the supplementary figure for Figure 3E and Figure 4C; the image presents the developing results of three parallel experiments.
**Figure S4:** High YTHDF3 expression is a protective factor in several cancers. (A–D) Survival analysis software package was used to analyse the survival prognosis difference between patients with high and low YTHDF3 expression in COAD, LAML, KIRC and MESO.

## Data Availability

The datasets used and/or analysed during the current study are available from the corresponding author on reasonable request.
