# Correction to ‘‘YTHDF3 Mediates the Occurrence and Development of Breast Cancer by Regulating Glycolysis Through the mTOR–HIF1α–LHDA Axis”

**DOI:** 10.1111/jcmm.71370

**Published:** 2026-10-06

Z. Liu , T. Jiang , P. Li , et al., “YTHDF3 Mediates the Occurrence and Development of Breast Cancer by Regulating Glycolysis Through the mTOR‐HIF1α‐LHDA Axis,” Journal of Cellular and Molecular Medicine 30, no. 9(2024), e18271, 10.1111/jcmm.71105.PMC1311141242036761

In Ziqian Liu et al., the representative experimental images for MCF‐7 cells shown in Figures 2C, F and G contain image misuse resulting from careless manual handling and technical error made during figure assembly. The correct figure is shown below. The authors confirm all results and conclusions of this article remain unchanged.

**FIGURE 2:**
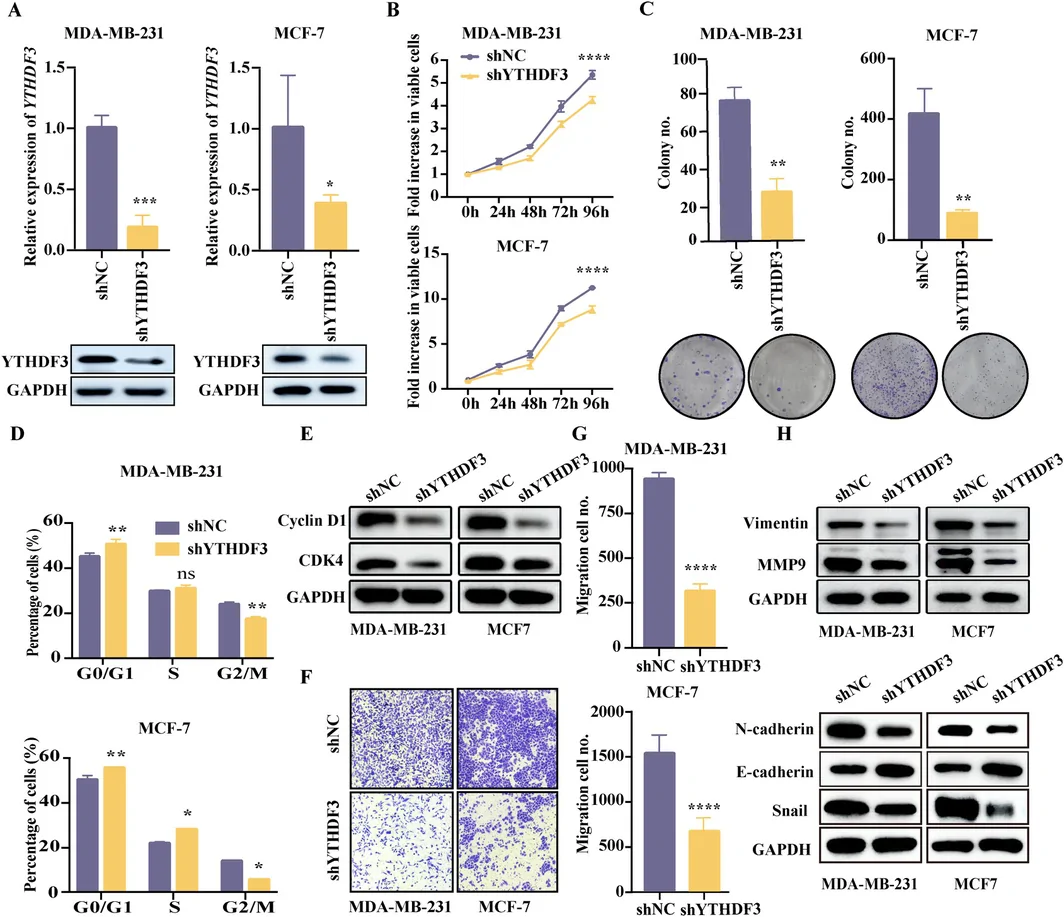
Silence of YTHDF3 restrain cell proliferation and migration of breast cancer cells and arrested the cell cycle. (A) Relative expression of YTHDF3 mRNA and protein after transfection of shNC and shYTHDF3 in MDA‐MB‐231 and MCF‐7 cells; (B) the growth ability of MDA‐MB‐231 and MCF‐7 cells transfected with shNC and shYTHDF3 were detected by CCK‐8 assay; (C) colony formation assay was used to detect the proliferation ability of MDA‐MB‐231 and MCF‐7 cells in shNC and shYTHDF3 groups; (D) the cell cycle distribution of MDA‐MB‐231 and MCF‐7 cells in shNC and shYTHDF3 groups was detected by flow cytometry; (E) expression of cyclin D1 and CDK4 in shNC and shYTHDF3 in MDA‐MB‐231 and MCF‐7 cells; (F, G) transwell invasion assay was used to detect the migration ability of breast cancer cells in the shNC group and the shYTHDF3 group in MCF‐7 and MDA‐MB‐231 cells; (H) the expression of vimentin, MMP9, E‐cadherin, N‐cadherin and snail in shNC and shYTHDF3 in MDA‐MB‐231 and MCF‐7 cells.

We apologize for this error.

