# Supplementary material for: YTHDF3 Mediates the Occurrence and Development of Breast Cancer by Regulating Glycolysis Through the mTOR–HIF1α–LHDA Axis
Source: J Cell Mol Med. 2026 Apr 26;30(9):e71105. doi: 10.1111/jcmm.71105 (PMC13111412; doi:10.1111/jcmm.71105)
Supplement: Supplementary file 1 — Figure S1: Expression of m6A ‘readers’ in cancer and adjacent tissues in the TCGA database. (A) YTHDF1 expression in breast cancer tissues and adjacent normal tissues in the TCGA database; (B) YTHDF2 expression in breast cancer tissues and adjacent normal tissues in the TCGA database; (C) YTHDC1 expression in breast cancer tissues and adjacent normal tissues in the TCGA database; (D) YTHDC2 expression in breast cancer tissues and adjacent normal tissues in the TCGA database. Figure S2: Silence of YTHDF3 restrains cell proliferation and migration of breast cancer cells and arrested the cell cycle. (A) Relative expression of YTHDF3 mRNA and protein after transfection of shNC and shYTHDF3‐2# in MDA‐MB‐231 and MCF‐7 cells; (B) the growth ability of MDA‐MB‐231 and MCF‐7 cells transfected with shNC and shYTHDF3‐2# was detected by the CCK‐8 assay; (C, D) colony formation assay was used to detect the proliferation ability of MDA‐MB‐231 and MCF‐7 cells in shNC and shYTHDF3‐2# groups; (E, F) transwell invasion assay was used to detect the migration ability of breast cancer cells in the shNC group and the shYTHDF3‐2# group in MCF‐7 and MDA‐MB‐231 cells. Figure S3: Supplementary experiments of YTHDF3. (A) The results of non‐mitochondrial, basal, maximal, proton leak, ATP production respiration and spare respiratory capacity OCR were normalized by protein amount (μg protein); (B) the mitochondrial complex I activity in the shNC group and the shYTHDF3 group; (C) the expression of YTHDF3 protein in shNC and shYTHDF3 groups of MDA‐MB‐231 and MCF‐7 cell lines; this is the supplementary figure for Figure 3E and Figure 4C; the image presents the developing results of three parallel experiments. Figure S4: High YTHDF3 expression is a protective factor in several cancers. (A–D) Survival analysis software package was used to analyse the survival prognosis difference between patients with high and low YTHDF3 expression in COAD, LAML, KIRC and MESO. [file JCMM-30-e71105-s001.docx]

Supplementary Figures and Legends


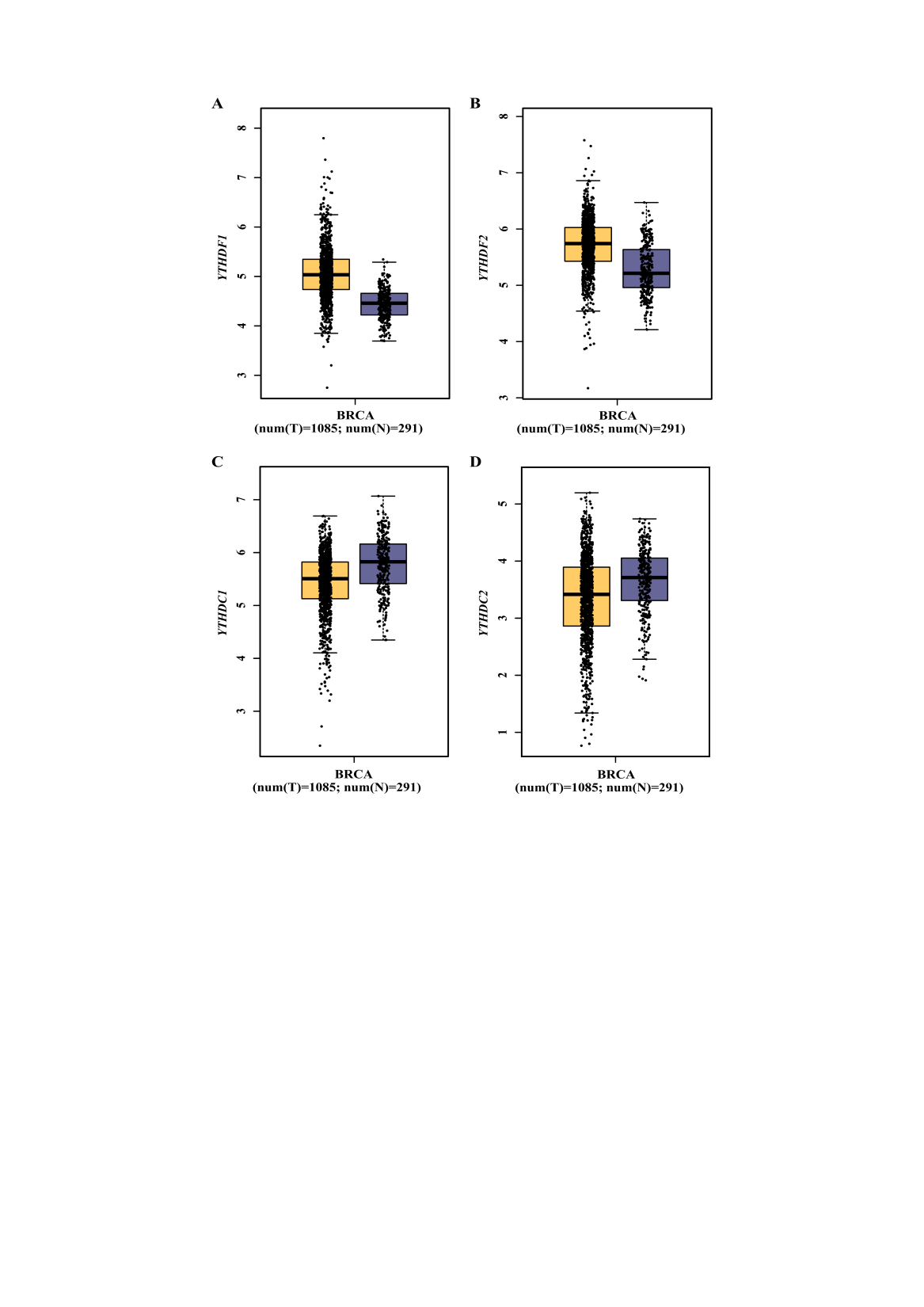


Figure S1. Expression of m6A "readers" in cancer and adjacent tissues in TCGA database. (a) YTHDF1 expression in breast cancer tissues and adjacent normal tissues in TCGA database; (b) YTHDF2 expression in breast cancer tissues and adjacent normal tissues in TCGA database; (c) YTHDC1 expression in breast cancer tissues and adjacent normal tissues in TCGA database; (d) YTHDC2 expression in breast cancer tissues and adjacent normal tissues in TCGA database.


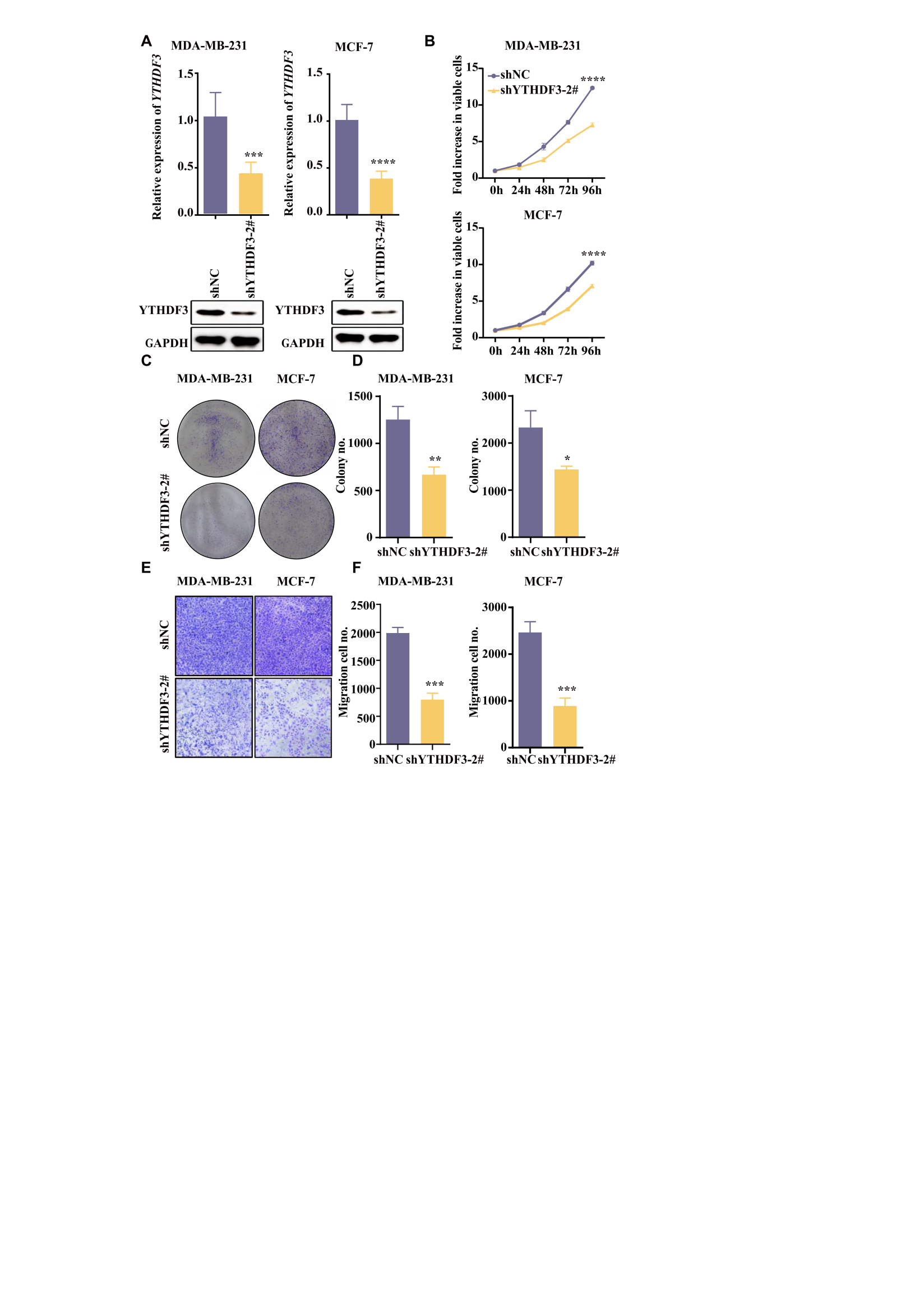


Figure S2. Silence of YTHDF3 restrain cell proliferation and migration of breast cancer cells and arrested the cell cycle. (a) Relative expression of YTHDF3 mRNA and protein after transfection of shNC and shYTHDF3-2# in MDA-MB-231 and MCF-7 cells; (b) The growth ability of MDA-MB-231 and MCF-7 cells transfected with shNC and shYTHDF3-2# was detected by CCK-8 assay; (c-d) Colony formation assay was used to detect the proliferation ability of MDA-MB-231 and MCF-7 cells in shNC and shYTHDF3-2# groups; (e-f) Transwell invasion assay was used to detect the migration ability of breast cancer cells in the shNC group and the shYTHDF3-2# group in MCF-7 and MDA-MB-231 cells.


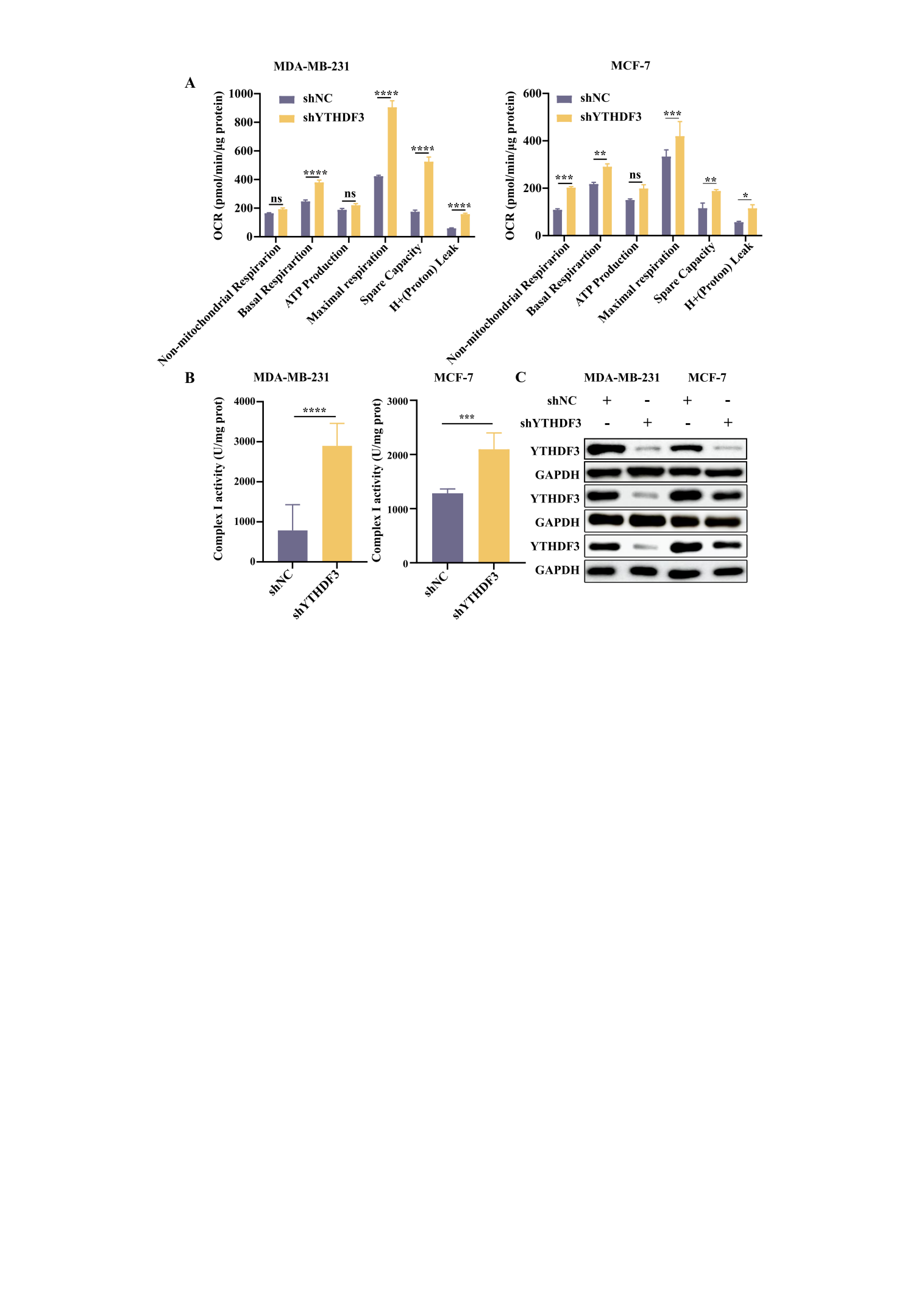


Figure S3. Supplementary experiments of YTHDF3. (a) The results of non-mitochondrial, basal, maximal, proton leak, ATP production respiration, and spare respiratory capacity OCR were normalized by protein amount (μg protein); (b) The mitochondrial complex I activity in the shNC group and the shYTHDF3 group; (c) The expression of YTHDF3 protein in shNC and shYTHDF3 groups of MDA-MB-231 and MCF-7 cell lines; This is the supplementary figure for Figure 3E and Figure 4C; The image presents the developing results of three parallel experiments.


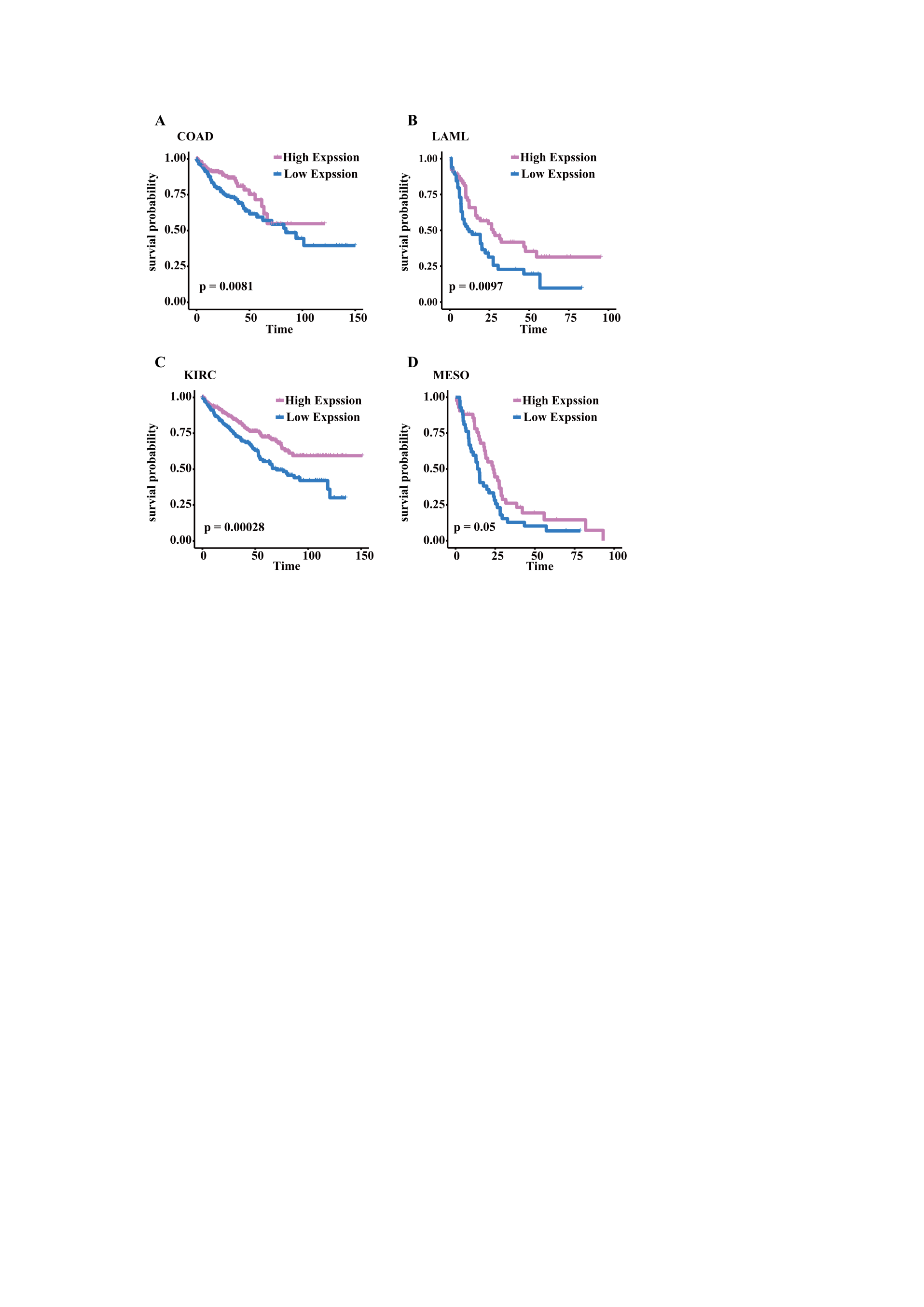


Figure S4. High YTHDF3 expression is a protective factor in several cancers. (a-d) Survival analysis software package was used to analyze the survival prognosis difference between patients with high and low YTHDF3 expression in COAD, LAML, KIRC, and MESO.
